# Regulation of Inflammatory Responses of Cow Mammary Epithelial Cells through MAPK Signaling Pathways of IL-17A Cytokines

**DOI:** 10.3390/ani14111572

**Published:** 2024-05-25

**Authors:** Kai Zhang, Min Zhang, Hong Su, Feifei Zhao, Daqing Wang, Yuanyuan Zhang, Guifang Cao, Yong Zhang

**Affiliations:** 1 College of Veterinary Medicine, Inner Mongolia Agricultural University, Hohhot 010011, China; zhangkai040423@163.com (K.Z.); zhangmin5400@126.com (M.Z.); hongsu1995@126.com (H.S.); imauzff@126.com (F.Z.); wangdaqing050789@126.com (D.W.); zyyworkaccount@163.com (Y.Z.); 2 Animal Embryo and Developmental Engineering Key Laboratory of Higher Education, Institutions of Inner Mongolia Autonomous Region, Hohhot 010011, China; 3 Inner Mongolia Autonomous Region Key Laboratory of Basic Veterinary Medicine, Hohhot 010011, China; 4 College of Life Sciences, Inner Mongolia University, Hohhot 010021, China

**Keywords:** cow mastitis, bacteria, infection, interleukin-17A

## Abstract

**Simple Summary:**

Cow mastitis is the most common and important economic problem among ruminant cows in the world, with bacterial infection constituting one of the main causes of the disease. Lipopolysaccharides (LPS) are one prototype Pathogen-Associated Molecular Pattern (PAMP) of Gram-negative bacteria and an important risk factor of cow mastitis caused by Gram-negative bacteria. Interleukin-17A (IL-17A), which is a signature cytokine of auxiliary T-cell 17, can be secreted by immune and non-immune cells. It has been proved that IL-17A plays a key role in the resistance of host animals against various bacterial infections. However, its role in the resistance of host animals against mastitis is not clear.

**Abstract:**

The aim of this study is to explore the mechanism of IL-17A infection in the development of bacterial mastitis in dairy cows. In this study, RT-qPCR and ELISA were used to measure the promoting effect of IL-17A on the generation of pro-inflammatory cytokines (TNF-α, IL-1β, and IL-6) and chemokine (IL-8). In addition, Western blot (WB) was applied to measure the influences of IL-17A on the inflammation-related ERK and p38 proteins in the MAPK pathways. The results show that under the stimulation of LPS on cow mammary epithelial cells (CMECs), cytokines IL-1β, IL-6, IL-8, TNF-α, and IL-17A will exhibit significantly increased expression levels (*p* < 0.05). With inhibited endogenous expression of IL-17A, cytokines IL-1β, IL-6, IL-8, and TNF-α will present reduced genetic expression (*p* < 0.01), with reduced phosphorylation levels of ERK and p38 proteins in the MAPK signaling pathways (*p* < 0.001). Upon the exogenous addition of the IL-17A cytokine, the genetic expression of cytokines IL-1β, IL-6, IL-8, and TNF-α will increase (*p* < 0.05), with increased phosphorylation levels of the ERK and p38 proteins in the MAPK signaling pathways (*p* < 0.001). These results indicate that under the stimulation of CMECs with LPS, IL-17A can be expressed together with relevant inflammatory cytokines. Meanwhile, the inflammatory responses of mammary epithelial cells are directly proportional to the expression levels of IL-17A inhibited alone or exogenously added. In summary, this study shows that IL-17A could be used as an important indicator for assessing the bacterial infections of mammary glands, indicating that IL-17A could be regarded as one potential therapeutic target for mastitis.

## 1. Introduction

Mastitis has a high prevalence rate among cows in the world, with 20 to 50 mastitis cases in every 100 cows annually [1]. The global concern about this problem is obvious because it not only incurs economic losses for dairy farms but also results in public health issues [2], making it one major endemic disease in the dairy industry. Although the world has taken positive measures to control cow mastitis, its incidence is still high, and it is still one of the highest incidence diseases in dairy cows. Among all pathogens causing cow mastitis, bacteria are one primary source of cow infections of clinical mastitis in the world [3]. Generally, this disease is caused by bacteria entering the internal cavities of cow mammary glands and settling in their epithelial barriers through nipple ducts [4]. Gram-positive bacteria, *Staphylococcus aureus*, and Gram-negative bacteria, *Escherichia coli*, are common bacteria that cause mastitis in cows [5]. The infection resistance capacities of cow breasts rely on the effectiveness of cow mammary immune systems, which cannot only prevent the invasion of bacteria into cow mammary glands but also eliminate existing infections and restore normal tissue functions [6].

Interleukin 17 (IL-17) is viewed as a key pro-inflammatory cytokine that plays a crucial role in the anti-tumor, anti-fungal, anti-bacterial, and anti-viral infections and inflammatory responses of organic bodies and is involved in the mediation of various autoimmune diseases [7,8]. The IL-17 family consists of six cytokines, which are IL-17A, IL-17B, IL-17C, IL-17D, IL-17E (IL-25), and IL-17F [9]. Among them, IL-17A is the most basic and most studied member of this cytokine family [10]. The general expression of IL-17R indicates that IL-17 can play a role in various cell types and tissues. However, the expression of IL-17 in mammary glands is little known. Some reports have shown that IL-17A can be used as an important indicator for assessing the bacterial infections of mammary glands [11], and there is IL-17A generated in mastitis caused by *streptococcus* or *S. aureus* infections [12,13,14]. Mammary epithelial cells are involved in the initial interactions between mastitis pathogens and host animals. Meanwhile, mammary epithelial cells can perceive the invasion of pathogens and react by activating their innate immune responses [15]. Indirect evidence has been reported that functional receptors of IL-17A are expressed in bovine mammary epithelial cells [16]. In addition, one study showed that IL-17A of recombinant cows can increase the production of TNF-α, IL-1β, and IL-6 in mammary epithelial cells of primary cows [14]. Rainard et al. argued that at the early stage of inflammation response, excessive genetic expression of IL-17A and IL-17F can be observed in RNA extracts of cow cells [11]. The results mentioned above indicate that IL-17A plays a crucial role in the inflammatory responses against bacterial infections and is closely associated with the incidence and development of bacterial mastitis among cows. However, the infection mechanism of IL-17A in the incidence and development of cow bacterial mastitis needs to be further investigated, attracting more and more attention from veterinarians and medical researchers.

In order to further explore the infection mechanism of IL-17A in the pathogenesis and development of bacterial mastitis in dairy cows, in this study, a mastitis model was constructed based on CMECs induced by LPS and the effect of IL-17A on the generation of pro-inflammatory cytokines (TNF-α, IL-1β, and IL-6) and chemokine (IL-8) was investigated. Furthermore, the influence of IL-17A on the activation of MAPK signaling pathways in CMECs was studied. A thorough investigation was conducted in this research on the mechanism of IL-17A involved in cow bacterial mastitis, providing a new potential therapeutic target and more valuable insights and help for disease prevention and control in the dairy cow production and breeding industry.

## 2. Materials and Methods

### 2.1. Reagents, Chemicals, and Antibodies

Fetal bovine serum (FBS), DMEM/F12 medium, and PBS (Hyclon, Logen, UT, USA); LPS (Sigma Aldrich, St. Louis, MO, USA); IL-17A (Peprotech, Cranbury, NJ, USA); Pierce BCA Protein Assay Kit (Beyotime, Shanghai, China); SDS-PAGE gelelectrophoresis kit (Solarbio, Beijing, China); Axy Prep Multisource Total mRNA Miniprep Kit (Axygen Scientific, Union City, CA, USA); SDS-PAGE Loading Buffer (Takara, Shiga, Japan); Primer Script RT Master Mix (Takara); SYBR Green Master (Rox) (Roche, Basel, Switzerland); M-PER mammalian protein extraction reagent (Thermo Fisher Scientific, Waltham, MA, USA); Phospho-ERK, ERK, Phospho-p38, p38, β-actin (Cell Signaling Technology, Beverly, MA, USA); Bovine IL-17A, TNF-α、IL-8、IL-1β、IL-6 ELISA kits (Qidibio, Wuhan, China); Halt Protease Inhibitor, Blocking Buffer, Pre-stained protein ladders, Starting Block T20 (Beyotime, Shanghai, China). All primers were synthesized by Sangon Biotech (Shanghai, China).

### 2.2. Isolation and Culture of CMECs

Healthy Holstein cows with an age of 6 years and a lactation period of about 90 days were selected, washed with 0.9% normal saline, and repeatedly washed with PBS containing 5% penicillin, streptomycin, and amphotericin, and the duct tissue, fat, and connective tissue were stripped. Catheter tissue, fat, and connective tissue were removed. After the cleaned tissue blocks were cut into fine pieces, 7–10 mL trypsin digestion solution (trypsin: collagenase I = 4:1) was added, and all of the pieces were sucked into a 15 mL centrifuge tube and digested on a shaking table at 37 °C for 1–3 h. When the tissue was fully digested, the centrifuge tube was removed and centrifuged at 1500 r/min for 5 min. The supernatant was absorbed, put into DMEM/F12 complete culture medium and filtered twice with a 400-mesh sieve. The filtered cells were centrifuged again, added to DMEM/F12 complete culture medium, mixed, and placed in a T25 cell culture bottle. It was placed in a 5% CO_2_ incubator at 37 °C until the cells grew to 80–90%, and then frozen storage, resuscitation, and subculture were performed.

### 2.3. Experimental Infection

All the cells used in the experiment were fifth-generation cells. In the LPS group, the supernatant was collected after 4, 8, 12, and 24 h of stimulation with a concentration of 10 μg·mL^−1^. The Y320 inhibitor group was added to the cells 24 h before and 48 h after the addition of LPS, and total RNA was extracted after the treatment of LPS for 12 h. Total RNA and total protein were extracted from cells of the IL-17A group after 4, 8, 12, and 24 h stimulation with a concentration of 100 ng·mL^−1^.

### 2.4. Enzyme-Linked Immunosorbent Assay (ELISA)

According to the instructions provided by each ELISA kit, different cytokines are detected, so that the corresponding standard curve is drawn and the concentration of each cytokine sample is calculated. Three biological replicates were performed for each factor.

### 2.5. Cell Viability Assay

Cell viability was determined by the CCK-8 method. CMECs were placed in 96-well plates with a density of 1 × 10^4^ cells per well and incubated at 37 °C with 5% CO_2_ for 24 h. Cells were incubated with designated concentrations of Y320 (10^−5^, 10^−6^, 10^−7^ M) for 4, 8, 12, 24, 48, and 72 h. The LPS group was incubated with specified concentrations (0, 1, 5, 10, 20, 50, 100 μg·mL^−1^) for a specified time (4, 6, 8, 12, 24 h). The IL-17A group was incubated with a specified concentration (0, 50, 100, 200 ng·mL^−1^) at a specified time point (3, 6, 8, 12, 24, 48 h). The cells were then incubated with 100 μL of fresh DMEM/F12 medium and 10 μL of CCK-8 solution (incubated in the dark for 4 h). Absorbance was recorded at 450 nm using an enzyme-labeled instrument. The experiment was repeated three times.

### 2.6. Western Blot Analysis

Total cell proteins were extracted on ice, cells were cleaved with cell lysate M-PER reagent, and protein concentrations were measured and decolorized according to the manufacturer’s instructions. Each lane was added with 20 μg of total protein, and electrophoresis was performed on SDS-PAGE (12%) at 80 V for 30 min, and 110 V for 1 h. The protein was transferred to the polyvinylidene fluoride (PVDF) membrane by using a semi-dry transmembrane at 25 V for 30 min. The membranes were blocked with TBST containing 3% BSA for 4 h at room temperature. The primary antibody was incubated at 4 °C (1:1000) for 14 h. The incubated film was washed with TBST 5 times for 5 min each time. The second antibody was incubated at room temperature for 1 h (1:3000). The membranes were then subjected to three 20 min TBST washes after incubation, and after that, exposed to electrochemiluminescence film and Western blot detection tools. Using ImageJ, band densities were measured.

### 2.7. Real-Time PCR Analysis

After LPS stimulate CMECs, total RNA is isolated. Total mRNA extraction and reverse transcription were performed. The polymerase chain reaction conditions were as follows: 50 °C, 2 min; 95 °C, 10 min; 95 °C, 15 s; and 60 °C, 60 s, for 40 cycles. The annealing temperature was 58 °C. The primers used in this study are listed in Table 1. The results were calculated using the 2^−∆∆Ct^ calculation method.

### 2.8. Statistical Analysis

All data in this paper were analyzed using GraphPad Prism 5 and expressed as mean ± standard deviation (SD). Statistical significance was evaluated using the mean ± SD of three independent experiments, and data was analyzed by one-way ANOVA followed by Tukey’s multiple comparisons test or 2-way ANOVA with Bonferroni’s post hoc test. A *p*-value ≤ 0.05 was considered statistically significant (ns *p >* 0.05, * *p* < 0.05, ** *p* < 0.01, *** *p* < 0.001).

## 3. Results

### 3.1. Influences of LPS on the Viability of CMECs and Generation of Pro-Inflammatory Cytokines, Chemokines, and IL-17A Cytokines

**Figure 1:**
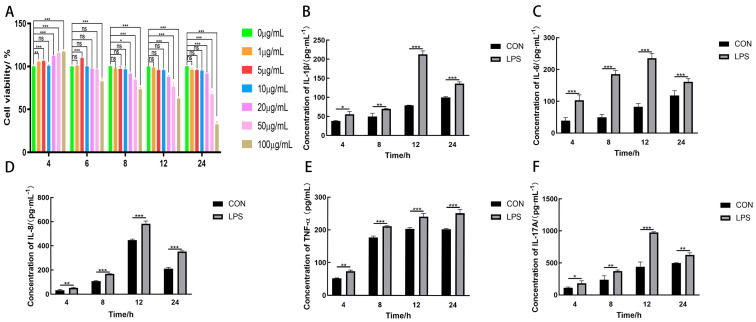
Effects of LPS on the activity of CMECs and the generation of pro-inflammatory cytokines, chemokines, and IL-17A cytokines. LPS with concentrations of 0 μg·mL^−1^, 1 μg·mL^−1^, 5 μg·mL^−1^, 10 μg·mL^−1^, 20 μg·mL^−1^, 50 μg·mL^−1^, and 100 μg·mL^−1^, and PBS (10 μL) were used to treat CMECs in all groups individually. Cell viability was measured using the CCK-8 method (**A**). After the treatment of CMECs with LPS (10 μg·mL^−1^) or not dealt with (CON), the concentrations of IL-1β, IL-6, IL-8, TNF-α, and IL-17A in the cell culture supernatant were measured using ELISA at time points of 4 h, 8 h, 12 h, and 24 h (**B**–**F**). Results are presented as the mean ± standard deviation of three sets of independent experimental data, with Bonferroni post hoc testing performed for two-factor analysis of variance (ns *p >* 0.05, * *p* < 0.05, ** *p* < 0.01, and *** *p* < 0.001).

A CCK-8 method was applied to assess the potential cytotoxicity of LPS on CMECs based on different time points (4, 6, 8, 12, and 24 h) and different concentrations (0, 1, 5, 10, 20, 50, and 100 μg·mL^−1^). Within a 24 h treatment period of LPS at a concentration range of 0–10 μg·mL^−1^, CMECs presented no significant changes in their viability, indicating that LPS exhibited no potential cytotoxic effect on CMECs within this time period and this concentration range (Figure 1A). Therefore, this concentration range can be used to measure the influence of LPS on CMECs within a treatment period of 24 h. In addition, the ELISA method was used to measure the influences of LPS (10 μg·mL^−1^) on the secretion status of IL-1β, IL-6, IL-8, TNF-α, and IL-17A from CMECs within a time period of 24 h. The results show that within time periods of 4, 8, 12, and 24 h, LPS can stimulate the secretion of IL-1β, IL-6, IL-8, and TNF-α from CMECs (*p* < 0.05; Figure 1B–E). Interestingly, it was found that IL-17A cytokines were also expressed in CMECs stimulated by LPS (*p* < 0.05; Figure 1F), with IL-1β, IL-6, IL-8, and IL-17A presenting the highest secretion levels at the time point of 12 h (*p* < 0.001) and TNF-α presenting the highest secretion level at the time point of 24 h (*p* < 0.001).

### 3.2. Regulation Effects of Endogenous IL-17A on the Generation of Pro-Inflammatory Cytokines and Chemokines in CMECs Stimulated by LPS

**Figure 2:**
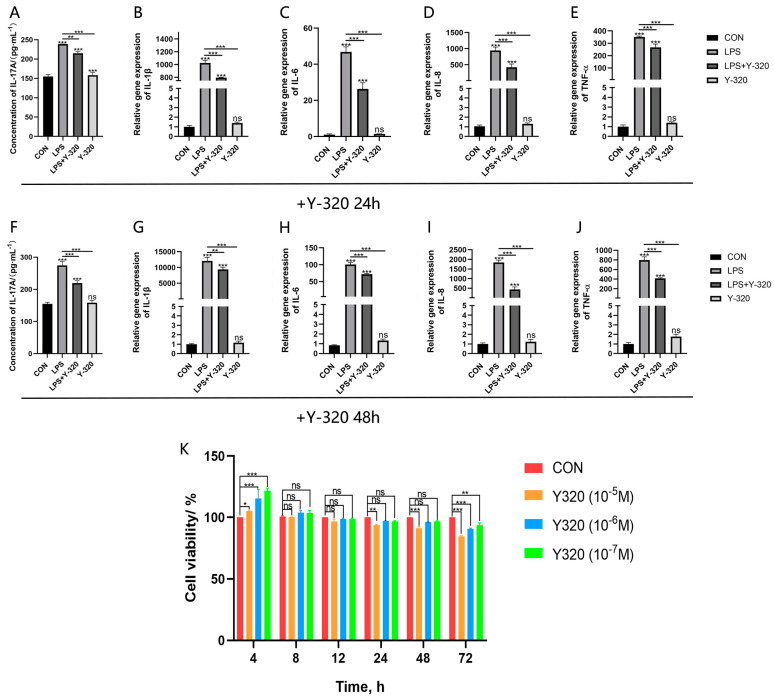
Regulation effects of endogenous IL-17A on the generation of pro-inflammatory cytokines and chemokines in CMECs stimulated by LPS. Y320 (10^−5^ M, 10^−6^ M, and 10^−7^ M) and PBS (10 μL) were used to treat CMECs in all groups individually. Cell viability was measured using the CCK-8 method (**K**). After 24 h and 48 h treatment periods of CMECs using Y320 (10^−6^ M) or not using any processing (CON), the concentrations of IL-17A in the cell culture supernatant were measured using ELISA (**A**,**F**). Genetic expression of IL-1β, IL-6, IL-8, and TNF-α was measured using RT-qPCR (**B**–**E**,**G**–**J**). Results are presented as mean ± standard deviation of three sets of independent experimental data, with Bonferroni post hoc testing performed for two-factor analysis of variance (ns *p* > 0.05, * *p* < 0.05, ** *p* < 0.01, and *** *p* < 0.001).

The CCK-8 method was used to evaluate the potential cytotoxicity of IL-17A inhibitor Y320 on CMECs at different time points (4, 8, 12, 24, 48, and 72 h) and different concentrations (10^−5^, 10^−6^, and 10^−7^ M). After a 48 h treatment period of Y320 at concentrations of 10^−6^ M and 10^−7^ M, CMECs presented no significant changes in their viability, indicating that Y320 exhibited no potential cytotoxic effects on CMECs within this time period and at these two concentrations (Figure 2K). Therefore, in this study, we selected a Y320 concentration of 10^−6^ M to inhibit the generation of IL-17A cytokines at two different time points of 24 h and 48 h, with the aim of evaluating the generation status of IL-17A, IL-1β, IL-6, IL-8, and TNF-α cytokines. The results show that after the treatment of IL-17A inhibitor Y320, CMECs stimulated by LPS present reduced secretion of IL-17A, IL-1β, IL-6, IL-8, and TNF-α (*p* < 0.01; Figure 2A–J), indicating that IL-17A cytokines may be involved in the genetic expression of IL-1β, IL-6, IL-8, and TNF-α.

### 3.3. Regulation Effect of Endogenous IL-17A on the Activation of MAPK Inflammatory Signaling Pathways in CMECs Stimulated by LPS

**Figure 3:**
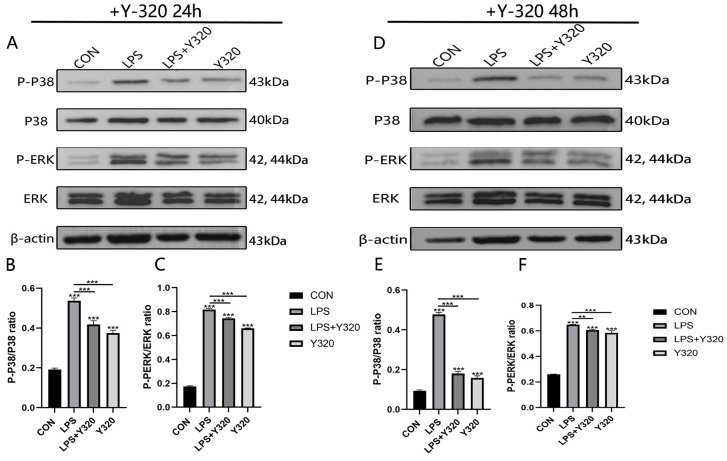
Regulation effect of endogenous IL-17A on the activation of MAPK inflammatory signaling pathways in CMECs stimulated by LPS. After 24 h and 48 h treatment periods of CMECs using Y320 (10^−6^ M) or not using any processing (CON), the phosphorylation levels of ERK and P38 in the MAPK signaling pathways of CMECs were measured using Western blot (**A**–**F**). Results are presented as mean ± standard deviation of three sets of independent experimental data, with Bonferroni post hoc testing performed for two-factor analysis of variance (** *p* < 0.01, and *** *p* < 0.001).

In this study, Y320 was used to inhibit the activation effects of the MAPK inflammatory signaling pathways at two different time points of 24 h and 48 h, with the aim of assessing whether endogenous IL-17A can regulate the activation of MAPK inflammatory signaling pathways. The results show that compared with CMECs stimulated by LPS, CMECs in the Y320 treatment group presented significantly down-regulated phosphorylation of ERK and p38 at time points of 24 h and 48 h (*p* < 0.001; Figure 3A–F). This indicates that during the stimulation of LPS to CMECs, endogenous IL-17A cytokines can affect the activation effect of MAPK signaling pathways.

### 3.4. Enhanced Genetic Expression of Pro-Inflammatory Cytokines and Chemokines in CMECs with Exogenous Addition of IL-17A

**Figure 4:**
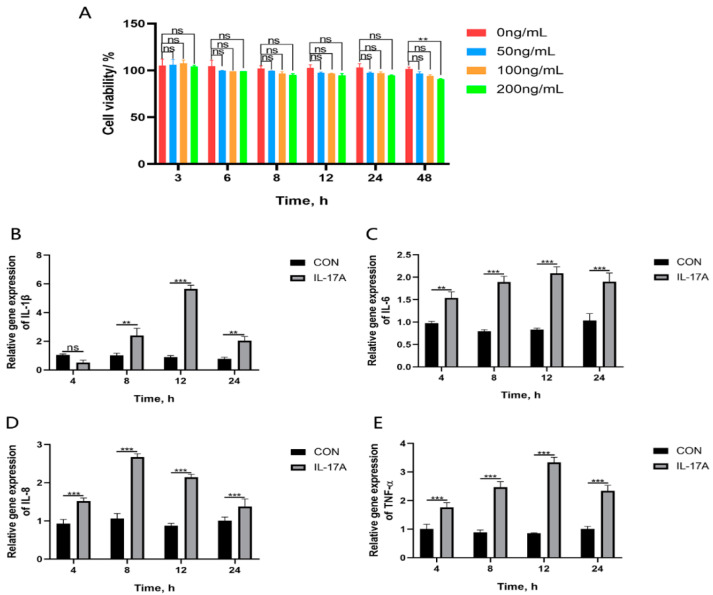
Enhanced genetic expression of pro-inflammatory cytokines and chemokines in CMECs with exogenous addition of IL-17A. IL-17A (50 ng·mL^−1^, 100 ng·mL^−1,^ and 200 ng·mL^−1^) and PBS (10 μL) were used to treat CMECs in all groups individually, with cell viability measured with the CCK-8 method (**A**). RT-qPCR was used to measure the concentrations of IL-1β, IL-6, IL-8, and TNF-α in the culture supernatant of CMECs (**B**–**E**). Results are presented as mean ± standard deviation of three sets of independent experimental data, with Bonferroni post hoc testing performed for two-factor analysis of variance (ns *p* > 0.05, ** *p* < 0.01, and *** *p* < 0.001).

The CCK-8 method was used to evaluate the potential cytotoxic effect of IL-17A on CMECs at different time points (3, 6, 8, 12, 24, and 48 h) and concentrations (0, 50, 100, and 200 ng·mL^−1^) selected in this study. The results show that after a 48 h treatment period of IL-17A with concentrations of 50 ng·mL^−1^ and 100 ng·mL^−1^, CMECs presented no significant changes in their viability, indicating that IL-17A exhibits no potential cytotoxicity on CMECs within this time period and at these concentrations (Figure 4A). Therefore, time points of 4 h, 8 h, 12 h, and 24 h were selected for using IL-17A (100 ng·mL^−1^) to stimulate the generation of CMECs or to not use any processing (CON), with the purpose of evaluating the influences of exogenous IL-17A on the generation of IL-1β, IL-6, IL-8, and TNF-α cytokines. The results show that after the treatment of IL-17A, the secretion amounts of IL-1β, IL-6, IL-8, and TNF-α in CMECs will be increased (*p* < 0.05; Figure 4B–E), with IL-1β, IL-6, and TNF-α presenting the highest genetic expression at 12 h and IL-8 presenting the highest genetic expression at 8 h (*p* < 0.001).

### 3.5. Activation of MAPK Signaling Pathways in CMECs through Exogenous Addition of IL-17A

**Figure 5:**
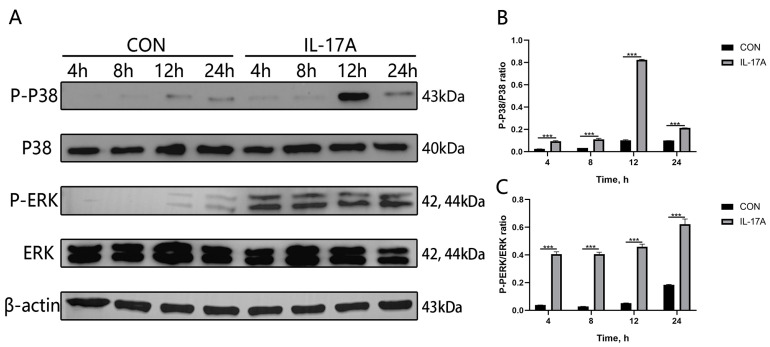
Activation of MAPK signaling pathways in CMECs with exogenous addition of IL-17A. After 4 h, 8 h, 12 h, and 24 h treatment periods of CMECs using IL-17A, the phosphorylation levels of ERK and P38 in the MAPK signaling pathways of CMECs were measured using Western blot (**A**–**C**). Results are presented as mean ± standard deviation of three sets of independent experimental data, with Bonferroni post hoc testing performed for two-factor analysis of variance (*** *p* < 0.001).

In this study, IL-17A (100 ng·mL^−1^) was used to stimulate CMECs or not using any processing (CON) at time points of 4 h, 8 h, 12 h, and 24 h to evaluate the influences of exogenous IL-17A on the phosphorylation levels of ERK and P38 in the MAPK signaling pathways of CMECs. The results show that compared with the blank control group, the IL-17A treatment group presents significantly down-regulated phosphorylation of ERK and p38 at time points of 4 h, 8 h, 12 h, and 24 h (*p* < 0.001; Figure 5A–C). It indicates that during the stimulation of IL-17A to CMECs, exogenous IL-17A can activate the MAPK signaling pathways.

## 4. Discussion

Over decades, many studies have been conducted on mastitis, which remains a primary problem in dairy farming. Cow mastitis is a common result caused by multiple factors such as heredity, environment, management, and nutrition [17]. Among all environmental factors, bacterial infection is the primary cause of mastitis [2,18], with such common bacteria as *S. aureus* and *E.coli* [5]. How to prevent and deal with cow bacterial mastitis is an important problem in public health. LPS are a major component of Gram-negative bacteria, causing similar inflammatory responses to those caused by intact *E.coli* [19]. Some studies have shown that IL-17A is an important effector of mammary immune responses to *E.coli*, and local enhanced production of IL-17A can improve the outcomes of early infections [20]. However, the specific function and mechanism of the IL-17A immune axis in LPS-induced cow mastitis remain unclear, and its function in the defense against mastitis needs to be further investigated.

An important aspect of the inflammatory response is the up-regulated secretion of cytokines and chemokines. For example, the excessive production of various cytokines and chemokines plays a vital role in the pathophysiology of septic shock caused by bacterial infections [19,21]. Studies by Ma et al. show that with the incidence of mastitis, the secretion levels of cytokines IL-1β, IL-6, and TNF-α will significantly increase, and IL-1β, IL-6, and TNF-α are important inflammatory cytokines that can trigger immune responses in organisms [22]. This study shows that under the stimulation of LPS, secretion amounts of relevant inflammatory cytokines IL-1β, IL-6, and TNF-α and chemokine IL-8 in CMECs will increase (Figure 1B–E), which is consistent with the study results of Ma et al. In addition, it was found that IL-17A cytokines are expressed in the inflammatory responses of CMECs induced by LPS (Figure 1F). The study results of Perrine et al. showed that genes encoding IL-17A in mammary glands during *E.coli* infections are overexpressed [23], which is consistent with our study results. This finding indicates that IL-17A may be used as a potential target for regulating the inflammatory responses of the mammary glands.

In order to further explore the role IL-17A plays in the inflammatory responses of CMECs, the IL-17A inhibitor Y320 was used to suppress the generation of endogenous IL-17A and evaluate whether IL-17A can regulate the inflammatory responses of mammary epithelial cells stimulated by LPS. The results show that after the addition of the Y320 inhibitor, IL-1β, IL-6, TNF-α, and IL-8 also present reduced genetic expression along with the reduced genetic expression of IL-17A. Some studies have shown that CMECs in culture respond to IL-17A through the overexpression of genes encoding IL-1β, IL-8, and IL-6 [9]. However, this study contradicts our results. We show that through the inhibition of IL-17A genetic expression, the genetic expression of IL-1β, IL-6, TNF-α, and IL-8 will also be inhibited (Figure 2). Furthermore, it has been found that relevant ERK and P38 proteins in the MAPK inflammatory signaling pathways present significantly reduced phosphorylation levels (Figure 3). The MAPK signaling pathway is a pathogenesis involved in immune responses and many inflammatory diseases [24]. This indicates that the genetic expression of IL-17A may be mediated through the MAPK signaling pathway. These aforementioned results show that the generation of the inflammatory cytokine IL-17A may be mediated through the regulation of the MAPK signaling pathway in the development of cow mastitis.

In order to further verify these results, exogenous IL-17A was added to act on CMECs. The results showed that the gene expression levels of cytokines IL-1β, IL-6, TNF-α, and IL-8 were increased after treatment of CMECs with IL-17A (Figure 4), and the phosphorylation levels of related ERK and P38 proteins in the MAPK inflammatory signaling pathway were also significantly increased (Figure 5). Hu et al. found that neutralizing antibodies for IL-17A can significantly inhibit the generation of inflammatory cytokines TNF-α and IL-1β and reduce LPS-induced pathological damage to mammary glands, and the neutralization of IL-17A could inhibit the generation of LPS-induced pro-inflammatory cytokines by inhibiting the activation of NF-κB signaling pathways [25]. This study is different from our study, which found that IL-17A may regulate the inflammatory response of dairy mammary epithelial cells through the MAPK signaling pathway. Hu et al. found that IL-17A regulates mastitis in dairy cows through the NF-κB signaling pathway. This provides a new idea and perspective for us to use to study IL-17A in bacterial cow mastitis in the future.

## 5. Conclusions

Based on this study, we found that not only TNF-α and IL-1β but also IL-6 and IL-8 can be regulated by IL-17A. Moreover, it was found that IL-17A can inhibit the generation of inflammatory cytokines induced by LPS through the activation of the MAPK signaling pathways. Therefore, based on all the data obtained in this study, it can be concluded that IL-17A can enhance the inflammatory responses of CMECs, thus providing a new potential therapeutic target for the treatment of cow mastitis.

## Figures and Tables

**Table 1:** Primer sequences for RT-qPCR.

Gene Name	Sequences (5′-3′)	Accession Number
β-actin	F:5′-CCAAGGCCAACCGTGAGAAGAT-3′ R:5′-CCACGTTCCGTGAGGATCTTCA-3′	NM_173979.3
IL-1β	F:5′-ATGAAGAGCTGCATCCAACACCTG-3′ R:5′-ACCGACACCACCTGCCTGAAG-3′	NM_174093.1
IL-6	F: 5′-ATGATGAGTGTGAAAGCAGCAAGG-3′ R:5′-TGATACTCCAGAAGACCAGCAGTG-3	NM_173923.2
IL-8	F: 5′-GCTGGCTGTTGCTCTCTTGG-3′ R: 5′-GGGTGGAAAGGTGTGGAATGTG-3′	NM_173925.2
TNF-α	F: 5′-CAACGGTGTGAAGCTGGAAGAC-3′ R:5′-TGAAGAGGACCTGTGAGTAGATGAG-3′	NM_173966.3

## Data Availability

Data are contained within the article.
